# Introducing a Rigid Loop Structure from Deer into Mouse Prion Protein Increases Its Propensity for Misfolding *In Vitro*

**DOI:** 10.1371/journal.pone.0066715

**Published:** 2013-06-25

**Authors:** Leah M. Kyle, Theodore R. John, Hermann M. Schätzl, Randolph V. Lewis

**Affiliations:** 1 Department of Molecular Biology, University of Wyoming, Laramie, Wyoming, United States of America; 2 Department of Veterinary Sciences, University of Wyoming, Laramie, Wyoming, United States of America; 3 Department of Biology and Synthetic Bioproducts Institute, Utah State University, Logan, Utah, United States of America; Ohio State University, United States of America

## Abstract

Prion diseases are fatal neurodegenerative disorders characterized by misfolding of the cellular prion protein (PrP^c^) into the disease-associated isoform (PrP^Sc^) that has increased β-sheet content and partial resistance to proteolytic digestion. Prion diseases from different mammalian species have varying propensities for transmission upon exposure of an uninfected host to the infectious agent. Chronic Wasting Disease (CWD) is a highly transmissible prion disease that affects free ranging and farmed populations of cervids including deer, elk and moose, as well as other mammals in experimental settings. The molecular mechanisms allowing CWD to maintain comparatively high transmission rates have not been determined. Previous work has identified a unique structural feature in cervid PrP, a rigid loop between β-sheet 2 and α-helix 2 on the surface of the protein. This study was designed to test the hypothesis that the rigid loop has a direct influence on the misfolding process. The rigid loop was introduced into murine PrP as the result of two amino acid substitutions: S170N and N174T. Wild-type and rigid loop murine PrP were expressed in *E. coli* and purified. Misfolding propensity was compared for the two proteins using biochemical techniques and cell free misfolding and conversion systems. Murine PrP with a rigid loop misfolded in cell free systems with greater propensity than wild type murine PrP. In a lipid-based conversion assay, rigid loop PrP converted to a PK resistant, aggregated isoform at lower concentrations than wild-type PrP. Using both proteins as substrates in real time quaking-induced conversion, rigid loop PrP adopted a misfolded isoform more readily than wild type PrP. Taken together, these findings may help explain the high transmission rates observed for CWD within cervids.

## Introduction

Prion diseases or transmissible spongiform encephalopathies (TSE's) are a class of fatal infectious neurodegenerative disorders [1]–[5]. Prion diseases are characterized by conformational conversion of the normal, cellular isoform of the prion protein (PrP^c^) into the disease-associated isoform (PrP^Sc^) which has an increased β-sheet content and a partial resistance to proteolytic digestion [6]–[8]. Different mammalian hosts are affected by various prion diseases, including scrapie in sheep and goat, bovine spongiform encephalopathy (BSE) in cattle, chronic wasting disease (CWD) in cervids, and Creutzfeldt-Jakob disease (CJD), Gerstmann-Straussler Scheinker disease (GSS), fatal familial insomnia (FFI), and kuru in humans. Transmission propensity upon exposure of a naïve host to prions varies for TSE's from different species and is modulated by species barrier and prion strain issues [9]–[11]. Compared to prion diseases in other species, CWD is highly transmissible [12]–[19]. CWD infectivity is found in a variety of tissues, body fluids and excretions, and is shed into the environment [12]–[19]. CWD is also the only prion disease that affects both free ranging and farmed animals, including deer, elk and moose, as well as other mammals in experimental settings [18], [19]. CWD poses currently insurmountable challenges to wildlife management strategies aimed at controlling the disease [18], [19]. While there have been recent gains in understanding about routes of transmission and the epidemiology of CWD, there has been little investigation into the molecular and cellular factors that may help CWD maintain such high transmission rates. [9], [18], [19].

Although both primary sequences and three-dimensional structures of PrP as determined by NMR are highly conserved among mammals [20], previous NMR work has identified a unique secondary structural feature in cervid PrP and PrP from a few other mammalian species, a rigid loop between β-sheet 2 and α-helix 2 on the surface of the molecule [21]. Although other amino acid substitutions provide loop rigidity in species such as the wallaby, horse, pig and rabbit [22]–[27], two amino acid substitutions in cervid PrP^c^ as compared to other species provides rigidity in the β-sheet 2 - α-helix 2 loop: S170N and N174T [21]. Introducing these two amino acid substitutions into murine PrP^c^ stabilizes its loop structure. Transgenic mice over-expressing mouse PrP^c^ with the two rigid loop associated substitutions developed spontaneous prion disease by an undetermined mechanism [28]. While the rigid loop region has been determined as an important determinant of the species barrier [14], [27], [29]–[31] and has been proposed to control susceptibility to prion disease [32], [33], its role in protein misfolding which is central to prion disease development has not been explored in full detail.

Our aim in this study was to test *in vitro* the hypothesis that the rigid loop has a direct influence on the prion protein misfolding process. Differences in misfolding with the introduction of the rigid loop could help to explain the high levels of transmission observed for CWD within cervid populations. Misfolding propensities for recombinant mouse PrP and a chimeric mouse/elk PrP with an introduced rigid loop were compared by biochemical techniques and cell free misfolding and conversion systems. We found that introducing the rigid loop increased the propensity of PrP to misfold in cell free systems.

## Methods

### Cloning and recombinant PrP expression

For expression of wild type mouse PrP (moPrP^WT^), PCR was used to amplify the coding sequence of mouse PrP (23–231). The murine *prnp* gene was inserted into pET30b. A plasmid for expression of mouse PrP with an introduced rigid loop (moPrP^RL^) was produced using the Quikchange site directed mutagenesis kit from Stratagene as per the manufacturer's recommendations. The S170N and N174T coding changes were introduced sequentially using the following primer pairs: GCCAGTGGATCAGTACAACAACCAGAACACCTTCG and CGAAGGTGTTCTGGTTGTTGTACTGATCCACTGGC (S170N), GTACAACAACCAGAACACCTTCGTGCACGACTGCG and CGCACGCGTGCACGAAGGTGTTCTGGTTGTTGTAC (N174T), and the moPrPWT pET30b plasmid as template. Rosetta (DE3) *E. coli* were used for expression of recombinant PrP (rPrP). Proteins were produced as previously described [34]. Briefly, cells were induced for 22–24 hours, using the Overnight Express Autoinduction System in LB media. Cells were harvested and subjected to two freeze/thaw cycles in liquid nitrogen before storage at −20°C. Before purification, inclusion bodies were washed twice with 0.1×BugBuster Master Mix (EMD), pelleted by centrifugation, and stored at −20°C. Alternatively, proteins were expressed in BL21 (DE3) *E. coli* using induction with 1 mM IPTG. Cells were lysed in lysis buffer (20 mM Tris HCl pH 7.4, 150 mM NaCl, 2 mM EDTA, 100 µg/mL lysozyme, 0.1% Triton X-100 and 10 mM β-mercapto-ethanol) and sonication (Misonix 3000 sonicator), followed by subsequent washes and pelleting by centrifugation in (20 mM Tris HCl pH 7.4, 2 M NaCl, 0.5% Triton X-100) containing 2, 4 and 6 M urea solutions. Purified inclusion bodies were then stored at -20°C. Proteins produced by this protocol were used for circular dichroism (CD) and for replications of the work presented here with the same conclusions. Both types of preparations were subjected to purification using the same protocol.

### Protein purification

Proteins were purified as previous described [34]. Proteins denatured in 8 M guanidine-HCl were incubated with 20 g Qiagen Ni-NTA superflow resin pre-equilibrated in denaturing buffer (100 mM sodium phosphate, 10 mM Tris, 6 M guanidine-HCl, pH 8.0). The slurry was loaded into an XK-16 column (GE Healthcare) for purification using an AKTA Explorer chromatography system at room temperature. Recombinant PrP was refolded on the column using a linear gradient of denaturing buffer to refolding buffer (100 mM sodium phosphate, 10 mM Tris, pH 8.0). Protein was eluted from the column using a linear gradient from refolding buffer to elution buffer (100 mM sodium phosphate, 10 mM Tris, 500 mM imidazole, pH 5.8). Eluted protein was collected in 2 ml fractions and diluted into 2 ml dialysis buffer (10 mM sodium phosphate, pH 5.8). rPrP containing fractions were pooled, 0.22 µm filtered, and then dialyzed into dialysis buffer using 7,000 MWCO SnakeSkin dialysis tubing (Pierce-Thermo) overnight at 4°C. Samples were filtered again following dialysis. Protein concentration was determined using a BCA Protein Assay Kit (Pierce-Thermo).

### Lipid vesicle preparation

1-palmitoyl-2-oleoyl-*sn*-glycero-3-phospho-(1′-*rac*-glycerol) (POPG) was purchased from Avanti Polar Lipids and dried at room temperature under a stream of nitrogen. Lipids were processed and stored under a nitrogen atmosphere. Lipids were rehydrated to a concentration of 2.5 mg/mL in 20 mM Tris-HCl, pH 7.4 and allowed to rehydrate at room temperature for a minimum of one hour before sonication in a cup horn using a Misonix 3000 sonicator until clear.

### Lipid based conversion assay, PK digestion and Western blot

For lipid based conversion assays, moPrP^WT^ or moPrP^RL^ were incubated with lipids or lipid free buffer (20 mM Tris-HCl, pH 7.4) at a ratio of 3 parts PrP to one part lipid or lipid free buffer in a volume to volume ratio for varying lengths of time. Proteinase K (PK) digestion was performed at a concentration of 5 µg/mL PK for 30 minutes at 37°C. The reaction was stopped using pefablock inhibitor and samples were centrifuged at 100,000×g for one hour (Beckman Optima TL ultra-centrifuge, TLA 45 rotor, 4°C). The resulting pellet was dissolved in 8 M urea and loading buffer. Samples not treated with PK were centrifuged as above. Both pellet fraction containing insoluble aggregates and the supernatant containing the soluble fraction of PrP were analyzed via SDS-PAGE and Western blot. The undigested pellet was dissolved in urea and loading buffer as above. All samples were boiled for five minutes previous to SDS-PAGE. Samples were run on 4–20% gradient gels from Pierce-Thermo and transferred to nitrocellulose membranes. Samples were probed with 4H11 anti-PrP monoclonal antibody at a concentration of 1∶5,000 for one hour. A goat, anti-mouse, alkaline phosphatase (AP) conjugated antibody from Promega was used as a secondary antibody. For visualization, BCIP/NBT from Pierce-Thermo was used as an AP substrate according to manufacturer's recommendations.

### Circular dichroism

Using a Jasco 720 circular dichroism (CD) spectrometer, far-UV spectra were obtained for moPrP^WT^ or moPrP^RL^. Lipid based conversion reactions and lipid free reactions were prepared as above. Measurements were obtained at a response time of 1 s, a resolution of 0.5 nm and standard sensitivity (100 mdeg). Ten scans were averaged for each protein and treatment. Readings were baseline subtracted, meaning that the background spectra from the buffer (buffer with or without lipid) was subtracted from the spectrum. Meaningful data could not be obtained below 200 nm due to high noise to signal ratio.

### Real time quaking-induced conversion (RT-QuIC)

RT-QuIC was performed as previously described [34]. Briefly, each reaction containing 0.1 mg/ml rPrP substrate, 20 mM sodium phosphate (pH 6.9), 300 mM NaCl, 1 mM EDTA, and 10 µM Thioflavin T were prepared in quadruplicate in a 96 well optical bottom plate (Nalge Nunc International). 10% w/v brain homogenates were prepared from the brains of mock infected and terminally ill 22L mouse adapted scrapie infected C57Bl/6 mice, mock infected and CWD infected transgenic mice expressing deer PrP^c^ (1536 Tg[CerPrP]) [35] and brain stem slices from uninfected white-tailed deer and white-tailed deer experimentally infected with CWD as described previously [36]. Ninety eight µL of substrate cocktail was seeded with 2 µl of brain homogenate serially diluted to various concentrations using 20 mM sodium phosphate (pH 6.9), 130 mM NaCl, 0.1% (w/v) SDS, 1X N2 Supplement (Invitrogen). The plate was sealed with Nunc Amplification Tape (Nalge Nunc International). Using a BMG Labtech FLUOstar Omega fluorescence plate reader pre-heated to 42°C, the reactions were subjected to a cycle of 1 min rest followed by 1 min shaking (700 rpm, double orbital) with fluorescence readings (450 nm excitation, 480 nm emission) every 15 minutes. This cycling was continued for 50 hours (200 readings). Data was plotted as the average of quadruplicate reactions using every third time point (45 min) with GraphPad Prism software. Time at beginning of conversion was determined as the time point at which fluorescence rose from baseline (17–20,000 relative fluorescence units) to above 24,000 fluorescence units. Time to start of conversion was analyzed statistically using GraphPad Prism software.

### Ethics statement

Original animal work that produced brain samples from terminal ill CWD infected and uninfected transgenic mice was performed at Lab Animal Resources, Colorado State University. Brains were kindly provided by Dr. M. Zabel and Dr. G. Telling. Mice were bred and maintained in animal facilities accredited by the Association for Assessment and Accreditation of Lab Animal Care International, in accordance with protocols approved by the Institutional Animal Care and Use Committee at Colorado State University. Intracerebral inoculations were performed under Isoflurane anesthesia, and mice euthanized using CO_2_ inhalation followed by decapitation. All efforts were made to minimize suffering.

## Results

### moPrP^RL^ converts to a lipid-induced, aggregated, proteinase K resistant isoform at lower concentrations than moPrP^WT^

It has been previously documented that anionic lipids can be used to stimulate spontaneous conversion and aggregation of rPrP expressed from a variety of mammalian genes in the absence of contact with infectious prions [37]–[40]. To investigate the role of the rigid loop on protein misfolding, we used a cell free system in which interactions with POPG lipid vesicles cause rPrP to undergo conformational changes to a PK resistant isoform. This method operates under physiological conditions in a simplified system and has been shown to produce misfolded rPrP with physical properties similar to naturally occurring prions [38]. Utilizing this method, we compared the propensity for misfolding *in vitro* in an unseeded manner for moPrP^WT^ and moPrP^RL^. Instead of comparing cervid rPrP with rPrP from another species such as murine rPrP, we decided to introduce the rigid loop (RL) structure into mouse PrP in order to be able to reduce observed effects to the RL structure alone. It was found that moPrP^RL^ converts at lower concentrations when incubated with POPG vesicles than moPrP^WT^.

**Figure 1:**
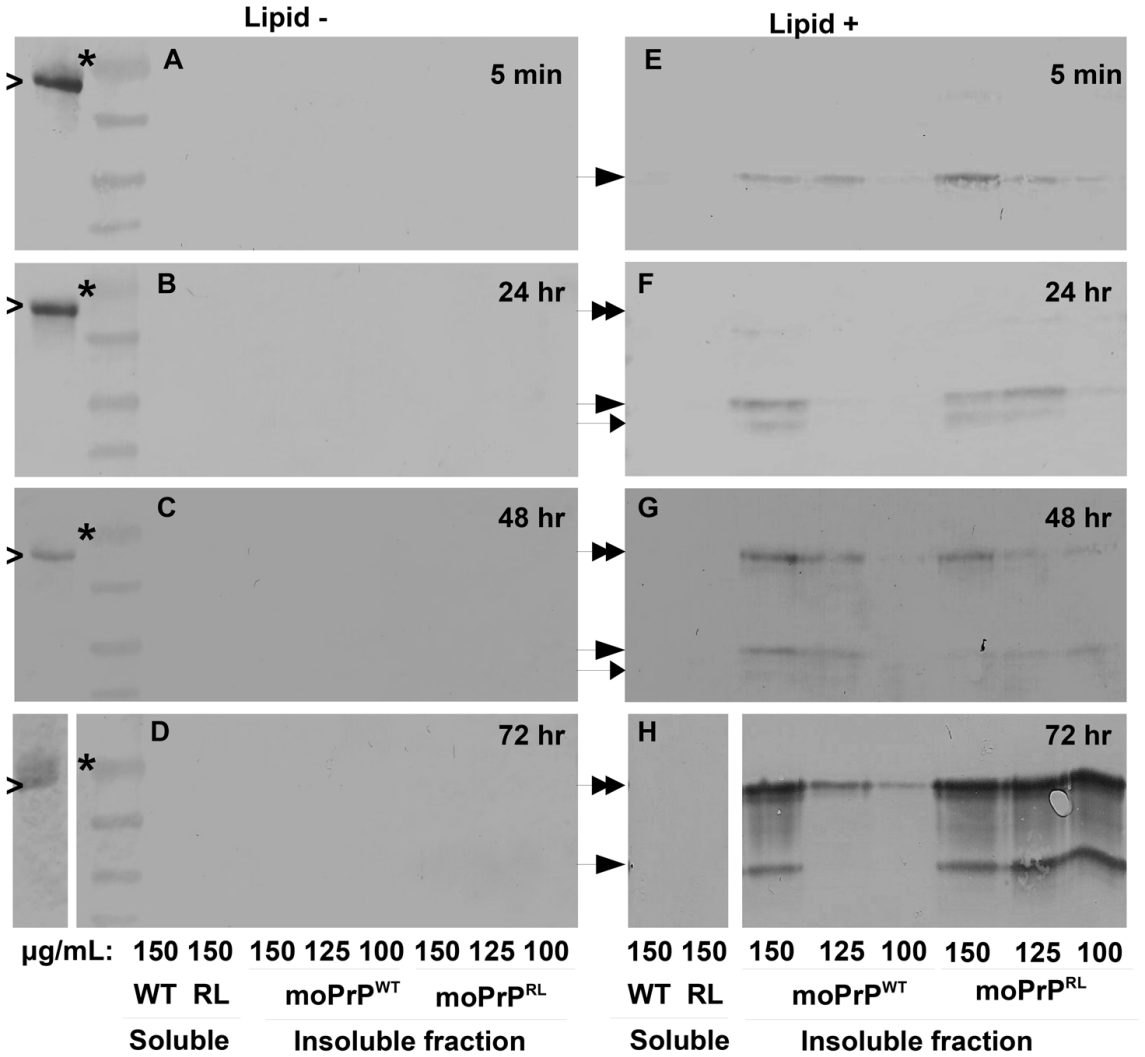
moPrP^RL^ converts to a PK resistant isoform at lower concentrations than moPrP^WT^. moPrP^WT^ and moPrP^RL^ at three different concentrations were incubated in the presence (E–H) or absence (A–D) of POPG lipid vesicles. Samples were removed for proteolytic digestion by proteinase K (PK) followed by ultra-centrifugation and Western blot analysis at four different time points. Generation of protease resistant, insoluble PrP was compared over time between moPrP^WT^ and moPrP^RL^. In POPG vesicle containing samples, generation of material that is both insoluble and resistant to PK digestion was detectible after only five minutes (E) as a 15 kDa PrP band (denoted by large arrow). A smaller molecular weight band of approximately 14.5 kDa (denoted by small arrow) was detectible by 24 hours (F). Conversion to a PK resistant, insoluble form at a concentration of 100 µg/mL is observed for only moPrP^RL^, consistently at all time periods analyzed (E–H, lanes 3 *vs* 6). Over time, PK treated, ultracentrifuged samples accumulated a 24 kDa band (denoted by double arrow) (E–H). This band may represent undigested rPrP or a dimer of smaller molecular weight, PK cleaved fragments. Generation of aggregated, PK resistant material in the absence of lipids was not detected for either moPrP^WT^ or moPrP^RL^ (A–D). PK resistant material was also undetectable in the soluble fraction (A–H). Data shown is representative of results from four independent experiments.

By Western blot analysis, aggregated, PK resistant isoforms were detectible for both moPrP^WT^ and moPrP^RL^ after treatment with POPG lipid vesicles, even after an incubation of only five minutes in duration (**Fig. 1** **E**). PK resistant material is observed by 14.5, 15 and 24 kDa signals, consistent with previous findings [38]. While the 14.5 and 15 kDa bands represent PK resistant core particles, the presence of the 24 kDa signal may result from one of two possibilities. The first possibility relies on the observation that lipids induce aggregation of rPrP [40]. As aggregation may shield full length rPrP from digestion by PK, it is plausible that full length protein remains undigested under conditions of lipid induced aggregation. Another explanation is that small molecular weight, PK resistant fragments form dimers as has been observed for newly converted misfolded isomers produced using QuIC [41]. Importantly, in the absence of lipids, no PK resistant, insoluble PrP was detectible (**Fig. 1** **A–D**), nor was PK resistant PrP detectible in the soluble fraction.

Previous studies have shown that POPG induced conversion is protein concentration dependent [38]. Therefore, we analyzed lipid based conversion at different rPrP concentrations. Differences were detected in amounts of aggregated, PK resistant material produced between the two proteins when reactions were assembled with decreasing protein concentrations (**Fig. 1** **E–H**). Using 125 and 100 µg/mL protein concentrations, moPrP^WT^ only poorly produced PK resistant fragments as compared to moPrP^RL^.

While both proteins were shown to convert to a PK-resistant, aggregated conformation, the observation that moPrP^RL^ converts at lower concentrations than moPrP^WT^ provides evidence that the rigid loop enhances PrP misfolding.

### Levels of lipid induced aggregated protein do not differ between moPrP^WT^ and moPrP^RL^

**Figure 2:**
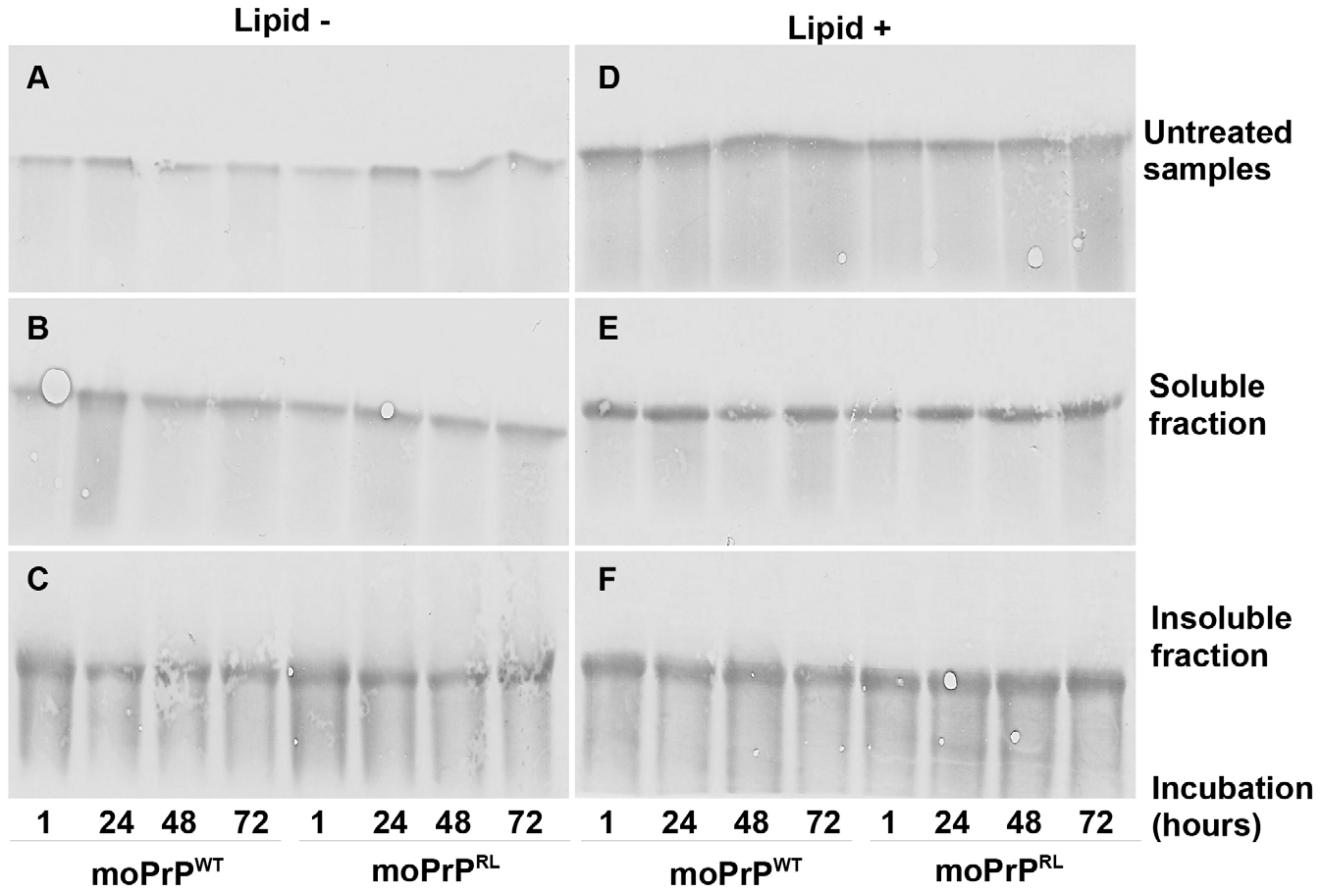
Altered lipid induced misfolding does not result from differences in aggregation between moPrP^WT^ and moPrP^RL^. Lipid- (left panels, A–C) and lipid+ reactions (right panels, D–F) containing 100 µg/mL of moPrP^WT^ or moPrP^RL^ were assembled such that all incubations were completed simultaneously. Untreated samples (A and D) were removed and the remaining sample was subjected to centrifugation to separate aggregated (C and F) from soluble PrP (B and E). Samples were then analyzed by Western blot analysis using mAb 4H11. Lipid- samples showed lower intensity than lipid+ samples despite equal starting concentration of protein reflecting that POPG lipid vesicles stabilize PrP at 37°C. moPrP^WT^ and moPrP^RL^ both showed equivalent amounts of soluble (B and E) and insoluble PrP (C and F), indicating that differences in lipid induced generation of PK resistant particles are obviously not the result of differing tendencies to aggregate between the two molecules.

Having seen that moPrP^RL^ converts to a PK resistant, aggregated form in the presence of POPG vesicles at lower concentrations than moPrP^WT^, we decided to compare levels of aggregated proteins between the two molecules. Basing our hypothesis on the previously published observation that aggregation leads to misfolding [40], we assessed the possibility that moPrP^RL^ undergoes lipid induced misfolding at lower concentrations because it has a greater tendency to aggregate than moPrP^WT^. To investigate this possibility, moPrP^WT^ and moPrP^RL^ samples incubated in the presence or absence of lipids were subjected to ultracentrifugation to separate aggregated from soluble, monomeric prion protein (**Fig. 2**).

After separation by ultra-centrifugation, differences were revealed between lipid containing reactions and reactions without lipids. When analyzed via Western blot, samples without lipids had consistently lower signal intensity than samples incubated in the presence of lipids, although both showed bands of the same molecular size (compare **Fig. 2** **A–C** and **Fig. 2** **D–F**). As conversion reactions with and without lipids had identical starting concentrations and were prepared from the same solutions, this finding suggests that rPrP is stabilized by the presence of POPG vesicles at 37°C. While differences were observed for lipid containing reactions and reactions without lipids, no differences were observed in amounts of soluble or aggregated protein between moPrP^WT^ and moPrP^RL^ conversion reactions, whether in the presence of lipids (**Fig. 2** **E–F**) or absence of lipids (**Fig. 2** **B–C**).

As levels of aggregation were comparable between moPrP^WT^ and moPrP^RL^ for a given treatment, we conclude that differences in aggregation are not the likely cause of differences observed in lipid based conversion between the two proteins.

### Lipid induced generation of PK-resistant, aggregated rPrP is accompanied by a shift in secondary structure toward increased β-sheet content

After analysis of lipid induced aggregation, we next verified that generation of PK resistant, aggregated rPrP is accompanied by a structural change indicative of protein misfolding. Previous studies have shown that association of rPrP with POPG vesicles induces a structural change from an α-helical conformation to a structure with increased β-sheet content [38]–[40]. To ensure that our experimental protocol produces PK resistant, aggregated rPrP with an altered conformation, lipid induced changes in secondary structure were investigated by far-UV circular dichroism (CD).

**Figure 3:**
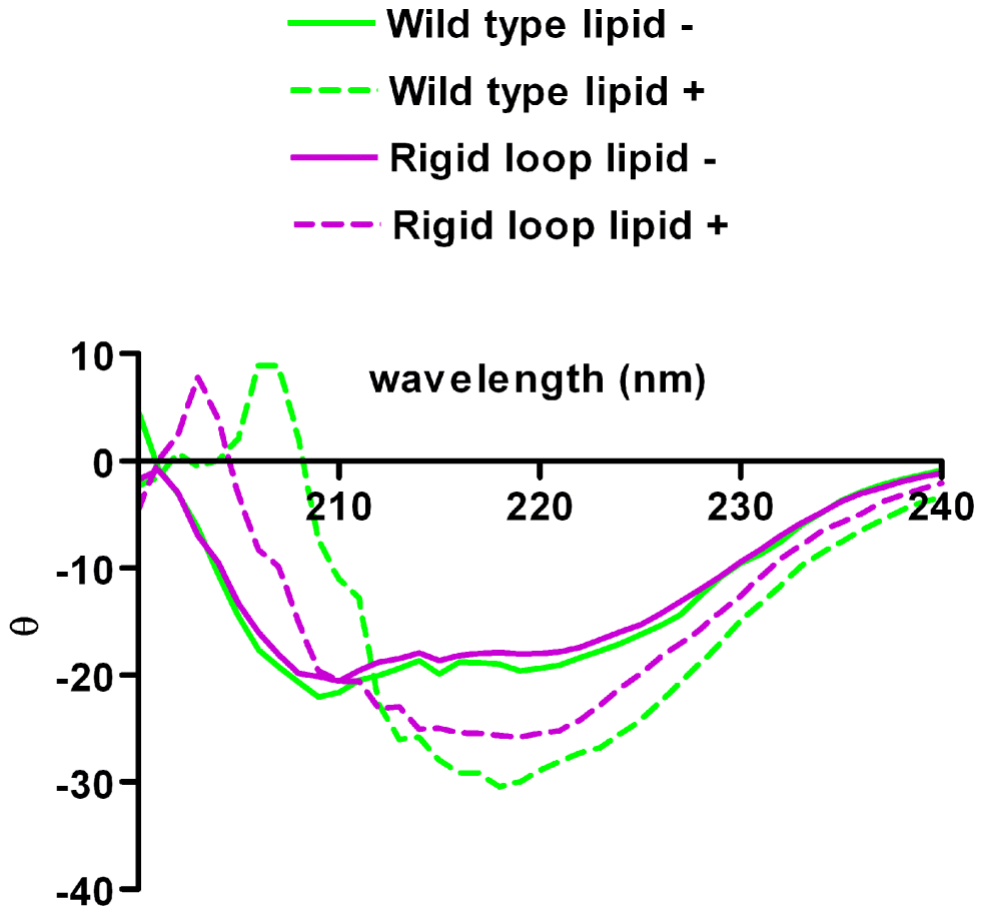
Generation of lipid induced, PK-resistant isoform is accompanied by a structural change increasing β-sheet content. Differences in secondary structure between moPrP^WT^ and moPrP^RL^ are not detectible by circular dichroism (CD) as shown by their nearly identical CD spectra (solid lines). Upon addition of POPG lipid vesicles, the secondary structure of both moPrP^WT^ and moPrP^RL^ changed to reflect an increase in β-sheet content (dashed lines). In the presence of lipids, moPrP^WT^ and moPrP^RL^ do not exhibit identical CD spectra (dashed lines), possibly indicating differences in lipid induced secondary structural changes between these molecules.

In analyzing secondary structure via CD, we found that both proteins shift from an α-helix enriched conformation in the absence of lipids to a conformation enriched in β-sheet content in the presence of lipids (**Fig. 3**). The spectra indicative of a conformation enriched in α-helices observed for both moPrP^WT^ and moPrP^RL^ without lipids are consistent with previous spectroscopic characterizations of rPrP [38], [42]. Also the shift in spectra observed for both molecules when in contact with POPG vesicles indicative of an increase in β-sheet content was as expected [38]–[40]. However, moPrP^WT^ and moPrP^RL^ did not exhibit identical spectra after lipid induced structural conversion.

Although the details of the different conformational state of moPrP^WT^ and moPrP^RL^ in the presence of lipids cannot be fully explored in the absence of higher resolution structural data, it implies that the rigid loop structural feature influences the outcome of lipid induced changes in secondary structure.

### Introduction of the rigid loop to moPrP^WT^ increases the speed at which it converts to a Thioflavin-T positive isoform in RT-QuIC

As it was found in unseeded conversion assays that introduction of the rigid loop increases the propensity of moPrP^WT^ to misfold, we next compared seeded conversion of moPrP^WT^ and moPrP^RL^ via RT-QuIC. Consistent with an increased propensity for misfolding seen in lipid induced, unseeded misfolding, moPrP^RL^ converted earlier than moPrP^WT^ to an isoform that binds Thioflavin-T, a dye that emits fluorescence after binding to amyloid-like structures.

**Figure 4:**
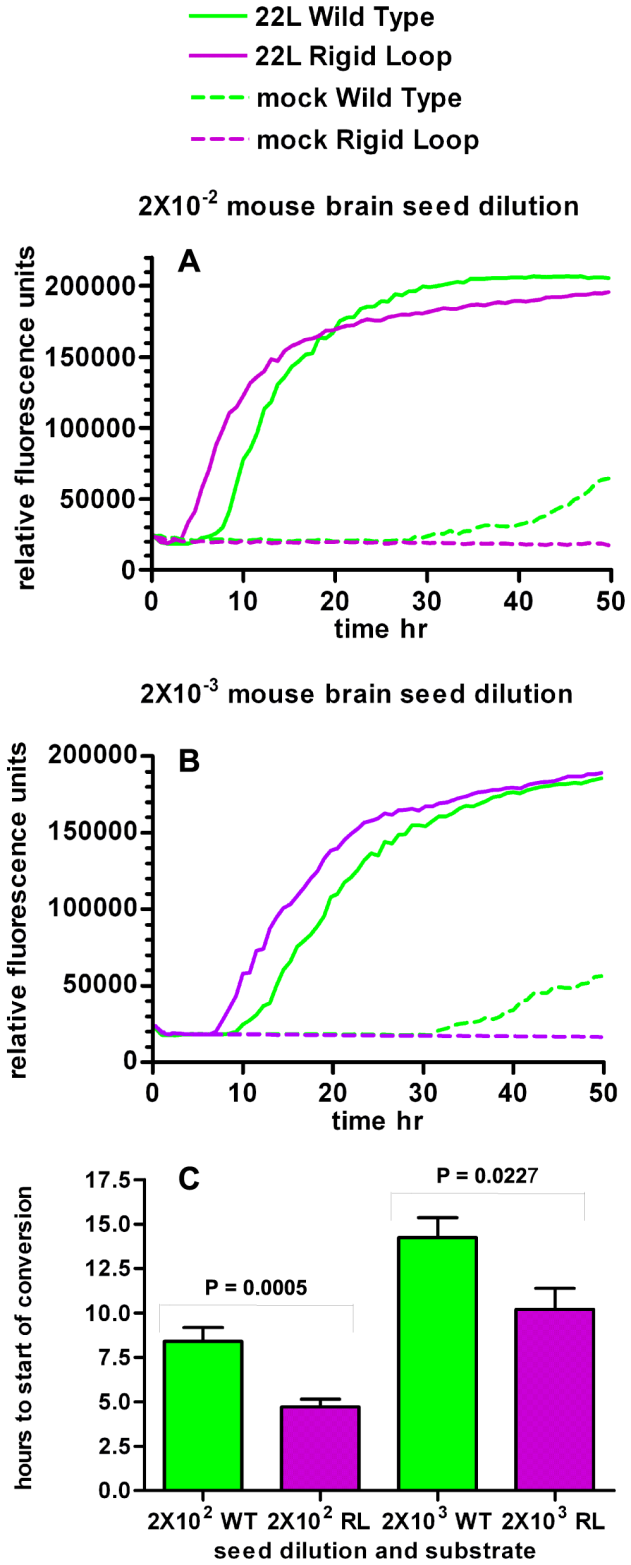
Generation of a Thioflavin T positive isoform via RT-QuIC occurs faster for moPrP^RL^ than moPrP^WT^. RT-QuIC reactions containing moPrP^WT^ (green lines) and moPrP^RL^ (purples lines) were seeded with brain homogenate from terminally ill 22 L prion infected mice (solid lines) or brain homogenate from mock infected controls (dashed lines) at a dilution of 2×10^−2^ (A) or 2×10^−3^ (B). Relative fluorescence over time was averaged for quadruplicate reactions. Time at beginning of conversion determined as the time point at which fluorescence rose from baseline to above 24,000 fluorescence units was compared for both dilutions of 22 L prion seed using GraphPad Prism software (shown in hours; data from 3 independent experiments) (C). Reactions with moPrP^RL^ as a substrate exhibited earlier conversion to a Thioflavin T positive amyloid structure than reactions with moPrP^WT^ substrate.

**Figure 5:**
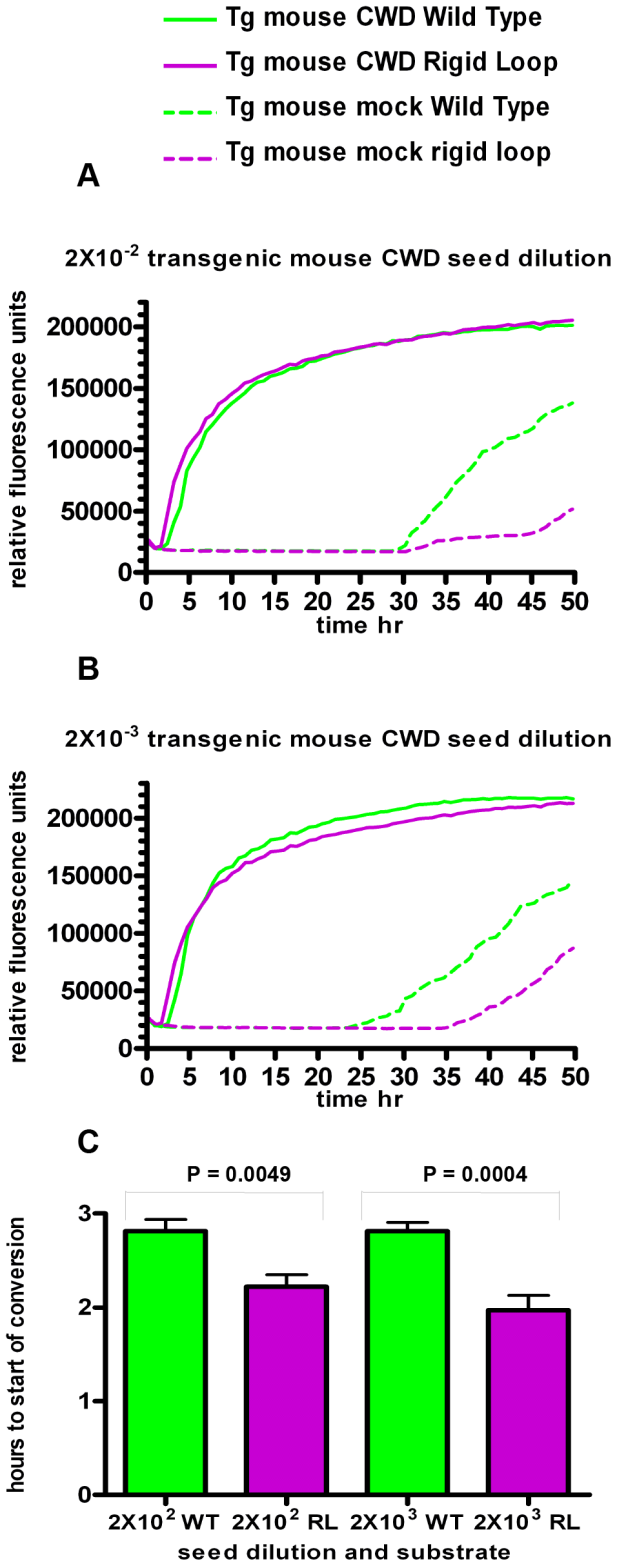
moPrP^RL^ converts faster than moPrP^WT^ in RT-QuIC reactions seeded with CWD prions from transgenic mice. RT-QuIC reactions containing moPrP^WT^ (green lines) and moPrP^RL^ (purples lines) were seeded with brain homogenate from terminally ill CWD prion infected transgenic mice expressing deer PrP^c^ (solid lines) or brain homogenate from mock infected control mice (dashed lines) at a dilution of 2×10^−2^ (A) or 2×10^−3^ (B). Data shown is the average of relative fluorescence over time for quadruplicate samples from two independent experiments. Reactions with moPrP^RL^ as a substrate exhibited earlier conversion when seeded with mouse CWD prions than reactions with moPrP^WT^ substrate (C).

**Figure 6:**
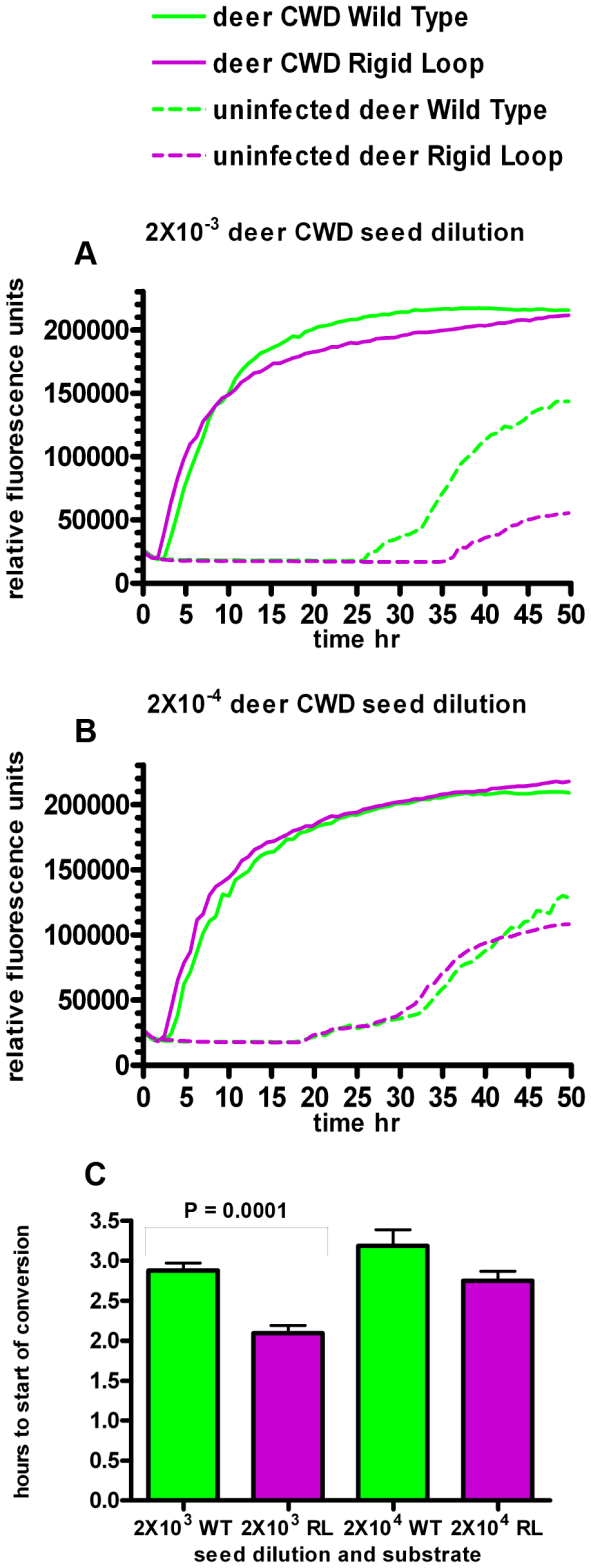
moPrP^RL^ converts faster than moPrP^WT^ in RT-QuIC reactions seeded with CWD prions from deer. RT-QuIC reactions containing moPrP^WT^ (green lines) and moPrP^RL^ (purples lines) were seeded with brain homogenate from terminally ill CWD prion infected white-tailed deer (solid lines) or brain homogenate from uninfected controls (dashed lines) at a dilution of 2×10^−3^ (A) or 2×10^−4^ (B). Reactions were prepared in quadruplicate and average fluorescence over time was averaged for two independent experiments (C). Reactions with moPrP^RL^ as a substrate exhibited earlier conversion than reactions with moPrP^WT^ substrate when seeded with CWD prions from the brain of an infected white-tailed deer.

RT-QuIC was developed as a highly sensitive assay for detection of infectious prions [34]. In this assay, prion sources of unknown titers are used to seed conversion of rPrP into a Thioflavin-T positive isoform through cycles of quaking and incubation [34]. Rather than using RT-QuIC to detect seeding differences between two sources of potential infectivity, RT-QuIC was utilized in this study to detect differences in misfolding between two different rPrP substrates when seeded with the same infectious prions. Generation of misfolded PrP is considered to be most efficient when there is a high level of primary sequence homology between infecting prions and the native PrP being recruited into a misfolded conformation. 22 L prions used here and moPrP^WT^ have identical primary amino acid sequences while moPrP^RL^ differs from the 22 L seed by two residues, S170N and N174T. Although moPrP^WT^ has greater sequence homology with 22 L prions, moPrP^RL^ converted to a Thioflavin-T positive isoform quicker than moPrP^WT^ when RT-QuIC reactions were seeded with brain homogenate containing 22 L mouse adapted scrapie prions (**Fig. 4**). Notably, faster misfolding was also observed for moPrP^RL^ when QuIC reactions were seeded with lower seed dilutions of 22 L and when reactions were seeded with other prion strains including CWD from cervidized transgenic mice (**Fig.5**) and CWD from white-tailed deer (**Fig. 6**).

In summary, introduction of the rigid loop results in earlier conversion of recombinant mouse PrP in real time quaking-induced conversion.

## Discussion

This study was designed to assess the influence of the rigid, β-sheet 2 - α-helix 2 loop from cervid PrP on prion protein misfolding. Using cell free conversion techniques, a key difference between moPrP^WT^ and moPrP^WT^ with an introduced rigid loop (moPrP^RL^) was identified. moPrP^RL^ was shown to convert to a misfolded isoform in cell free systems more readily than moPrP^WT^. Both unseeded conversion via association with POPG lipid vesicles and seeded conversion using RT-QuIC were utilized in this study. The use of RT-QuIC to explore differences in conversion dynamics between two PrP variants is a novel application for QuIC which was developed originally as a diagnostic technique. Chronic wasting disease is highly transmissible among cervids [12]–[19] and the finding that introduction of the rigid loop increases the propensity by which moPrP^WT^ misfolds could be relevant to the high transmission and shedding rates observed for CWD.

In investigating the role of the rigid loop on prion protein misfolding, we opted to study murine PrP with an introduced rigid loop rather than wild type cervid PrP. Comparative analysis of moPrP^WT^ and moPrP^RL^ allowed us to directly draw conclusions on the influence of the rigid loop on PrP misfolding without the need to detangle it from the influence of other amino acid variations observed for cervid PrP and species barrier issues. Importantly, the use of moPrP^RL^ allowed the use of moPrP^WT^ as a control. This study would have lacked this critical control if the experiments were performed with cervid PrP. As such, we were able to determine that the introduction of the rigid loop increases the propensity for moPrP^WT^ to misfold in both seeded and unseeded conversion reactions.

Although both the S170N and N174T substitutions are required for full stabilization of the β-sheet 2 - α-helix 2 loop region, there is evidence from NMR and modeling studies that S170 is the primary determinant of mobility of the loop region between β-sheet 2 and α-helix 2 [21], [43]. In simulations, mobility of the loop was noted to disrupt hydrophobic stacking interactions as well as hydrogen bonds that help to connect separated regions of PrP, potentially facilitating conformational change to a isoform with increased β-sheet content that was observed during simulations using the moPrP^WT^ sequence [43]. Lack of mobility in the loop region improved the electrostatic interaction energy of the molecule in simulations [43]. Additionally, NMR calculations indicated slightly higher stability for moPrP^RL^ than moPrP^WT^ [21] and moPrP^RL^ has been reported to be less thermally labile [33].

It has long been proposed that reduced structural stability of prion proteins is associated with increased propensity for misfolding. This is supported by the observation that some mutations in human *PRNP* which are associated with familiar forms of prion diseases disrupt salt bridges, alter hydrophobic reactions or otherwise reduce the global stability of PrP^c^ [44]–[47], and that partial denaturation of PrP stimulates misfolding *in vitro* [33], [48]–[54]. As such, it would be expected that moPrP^WT^ would exhibit a greater misfolding propensity than moPrP^RL^ as the rigid loop *per se* increases the structural stability of the molecule. In our study, introduction of the rigid loop clearly enhanced the propensity of the resulting recombinant protein to misfold *in vitro*. Although structural instability is sometimes correlated with misfolding, there is evidence that familial prion diseases cannot entirely be defined by thermodynamic destabilization of human PrP^c^ by amino acid mutations [47], [55], [56]. Some of the disease-associated mutations in humans and polymorphisms in animals that modulate susceptibility to disease do not alter the thermodynamic stability of PrP^c^ or are even associated with increased stability [32], [33], [47], [55]–[61]. Furthermore, increased stability has been observed to correlate to increased misfolding *in vitro* [32], [33]. Further investigation of the influence of structural stability on misfolding of cervid PrP molecules as compared to those from other species may aid in understanding the mechanism by which the rigid loop structure enhances misfolding in our *in vitro* system.

As differences in structural stability may not be the only explanation for enhanced misfolding as observed for moPrP^RL^, we next investigated whether introduction of the rigid loop altered lipid induced aggregation of moPrP^WT^. Enhanced aggregation upon the introduction of the rigid loop has previously been suggested as a possible explanation for development of *de novo* prion disease in mice over-expressing mouse/elk chimeric PrP with an introduced rigid loop [28]. In our *in vitro* studies, we did not detect differences in lipid induced aggregation between moPrP^WT^ and moPrP^RL^. Therefore, we do not consider differences in aggregation as the likely cause of increased misfolding propensity observed here *in vitro* for moPrP^RL^.

Although a mechanistic explanation of enhanced misfolding upon the introduction of the rigid loop has yet to be determined, we conjecture that perhaps the rigid loop confers differences in ability to form lipid associations in a manner that enhances misfolding. There is increasing evidence that lipids play a direct role in prion protein misfolding [6], [39], [40], [51], [62]–[66], perhaps functioning as a lipo-chaperone modulating the conformation of PrP [48]. Our conversion assays heavily utilized lipids (POPG in lipid based conversion assays and brain derived lipids in RT-QuIC). A conformation with increased β-sheet content is expected to be a thermodynamically favorable state for rPrP [48], [50], [67] and it has long been suggested that unknown cofactors could lower the activation energy for misfolding, thus enhancing misfolding [33], [67]–[69]. PrP^c^ variants could differ in conversion efficiency as a result of alterations in the ability of cofactors to modulate stability and folding [33]. Alterations in surface charge distribution have been proposed to alter interactions with entities that can modulate misfolding such as membrane lipids and other potential cofactors [44], [56]. Therefore, it is plausible that moPrP^RL^ exhibits a greater propensity for misfolding than moPrP^WT^ due to alterations in interactions with critical lipid cofactors. There is experimental evidence that the rigid loop region along with the C-terminal portion of helix 3 are the putative site for cofactor binding [68], [70], which adds support to this hypothesis.

Transmission studies on mice expressing wild-type and mouse/elk chimeric PrP have provided evidence that the β-sheet 2 – α-helix 2 loop region is also an important determinant for the species barrier [14], [27], [29]–[31]. Further support for this theory has been provided by studies using protein misfolding cyclic amplification (PMCA) [27], [31]. With few exceptions, it was observed that prions with rigid loop residues 170N and 174T recruit PrP^c^ into a misfolded form if the PrP^c^ also has the rigid loop, while prions without the rigid loop variations do not [14], [27], [29]–[31]. The reverse has also been found: prions without the rigid loop substitutions convert PrP^c^ that also lacks the substitutions while prions with the rigid loop do not [14], [27], [29]–[31]. Slightly in contrast with these previous findings, moPrP^RL^ misfolded earlier than moPrP^WT^ in our study when RT-QuIC reactions were seeded with 22 L mouse adapted scrapie prions. Earlier conversion for moPrP^RL^ occurred even though the 22 L seed prions lack the rigid loop associated variations. However, classical species barriers based on PrP homology are not consistently preserved in *in vitro* conversion systems such as RT-QuIC. Notably, the failure of our experimental system to recapitulate previously observed species barriers strengthens our argument that the enhanced misfolding propensity of moPrP^RL^ is based on an intrinsic structural feature. Nevertheless, we wanted to verify our findings using other prion strains. Earlier conversion for moPrP^RL^ than for moPrP^WT^ was consistent when RT-QuIC reactions were seeded with 22 L mouse prions, CWD prions from brains of transgenic mice as well as CWD prions from infected deer brain. Stronger conversion of moPrP^RL^ by CWD prions was anticipated based on previous studies. However, stronger conversion of moPrP^RL^ when seeded with 22 L prions was not anticipated. The finding that moPrP^RL^ converted more readily when seeded with 22 L prions although moPrP^WT^ has identical primary sequence homology and a homologous loop structure with 22 L prions is a very strong indication that introduction of the rigid loop increases the propensity of moPrP^WT^ to misfold.

In summary, introduction of the rigid loop, a structure inherent to cervid PrP, increases the propensity for recombinant mouse PrP to misfold. This was assessed *in vitro* using both an unseeded, lipid based conversion assay and seeded conversion using RT-QuIC to detect differences in conversion rates between two PrP variants. This finding may have relevance to the high levels of shedding of CWD prions and transmission of CWD among cervids as compared to prion diseases in other species.
